# Is androgen receptor activity in metastatic prostate cancer a good biomarker for bipolar androgen therapy?

**DOI:** 10.1172/JCI165357

**Published:** 2022-12-01

**Authors:** Nahuel Peinetti, Marijo Bilusic, Kerry L. Burnstein

**Affiliations:** 1 Department of Molecular and Cellular Pharmacology, University of Miami Miller School of Medicine, Miami, Florida, USA.; 2 Sylvester Comprehensive Cancer Center, University of Miami, Miami, Florida, USA.; 3 Department of Medicine, Division of Medical Oncology, University of Miami Miller School of Medicine, Miami, Florida, USA.; 4 Department of Veterans Affairs, Miami, Florida, USA.

## Abstract

Androgen deprivation therapy (ADT) is the longstanding treatment for advanced prostate cancer (PC) because androgen receptor (AR) is the key therapeutic vulnerability for this disease. Bipolar androgen therapy (BAT) — the rapid cycling of supraphysiologic androgen (SPA) and low serum testosterone levels — is an alternative concept, but not all patients respond and acquired resistance can occur. In this issue of the *JCI*, Sena et al. developed a gene signature indicative of high AR activity to predict patient response to BAT, including a decline in both serum prostate-specific antigen (PSA) and tumor volume. Preclinical models showed that AR-mediated suppression of MYC, known to drive PC, was associated with decreased cell growth following SPA treatment. Because BAT eventually leads to resistance, the authors tested cycling between SPA and AR antagonism in a patient-derived xenograft and observed a delay in tumor growth. These findings represent a major step toward the informed use of BAT for advanced PC.

## Overcoming castration resistance using supraphysiologic androgens

Because androgen receptor (AR) signaling promotes growth and survival of prostate cancer (PC), blocking AR activation has been the cornerstone of treatment for more than 70 years. Androgen deprivation therapy (ADT) decreases the synthesis of the two major AR ligands, testosterone and dihydrotestosterone (DHT) using luteinizing hormone-releasing hormone (LHRH) agonists or LHRH antagonists. Despite initial therapeutic benefits, the disease inevitably progresses to an incurable stage termed castration-resistant PC (CRPC). This ultimate stage arises through a variety of mechanisms that promote persistent or reactivated AR signaling (reviewed in refs. 1, 2). Many men with CRPC respond to newer generation AR signaling inhibitors (ARSIs) such as the AR antagonists enzalutamide, apalutamide, and darolutamide, or the androgen biosynthesis inhibitor abiraterone acetate, but inevitably progress within one to two years (2). Thus, the discovery that supraphysiologic concentrations of androgens (SPA) could block the growth of PC was seemingly paradoxical. However, the well documented but incompletely understood phenomenon in which PC cell proliferation is stimulated by low — but inhibited by high — androgen levels provided a foundation for the use of SPA therapy (reviewed in ref. 3).

SPA treatment, similar to ADT and other targeted therapies, ultimately results in therapeutic resistance for preclinical CRPC models and PC patients (4). To overcome tumor adaptation to high testosterone levels, Samuel Denmeade and John Isaacs pioneered bipolar androgen therapy (BAT) in which SPA is pulsed with continuous ADT in order to cycle serum testosterone between high — supraphysiologic — and low — castrate — levels (4). Several clinical trials have demonstrated that BAT is safe in asymptomatic patients and can produce durable prostate specific antigen (PSA) and objective responses in approximately 30%–40% of PC patients (5–9). Since not all patients respond to BAT — as determined by decreased serum PSA or tumor volume, or by longer radiographic progression-free survival — and the degree and durability of response are unpredictable, identification of prognostic and predictive biomarkers is needed (10, 11).

## High AR activity is associated with clinical response to BAT

In this issue of the *JCI*, Sena et al. leveraged sequential paired metastatic specimens, termed “pre-BAT” and “on-BAT”, that were collected from an ongoing clinical trial COMBAT-CRPC (NCT03554317). This single arm, phase II clinical trial enrolled patients with metastatic CRPC whose cancer progressed on an ARSI and treated them with BAT for 12 weeks, followed by a combination treatment of BAT and the anti-PD1 agent nivolumab (12). Specimens for this study were collected prior to the initiation of treatment with nivolumab.

**Figure 1:**
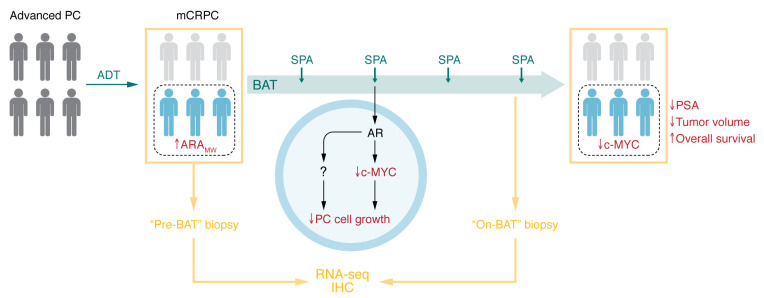
ARA_MW_ is a potential biomarker of BAT response in metastatic CRPC. Patients with advanced PC are commonly treated with ADT but generally progress to incurable CRPC. The COMBAT-CRPC clinical trial enrolled patients with metastatic CRPC (mCRPC) and treated them with BAT for 12 weeks with three cycles of SPA with ongoing ADT. Sena et al. performed RNA-Seq and IHC analysis of metastatic tumor biopsies obtained from patients before treatment and after the three cycles of BAT. The authors used RNA-Seq data to demonstrate that high ARA_MW_ in pretreatment metastatic biopsies was associated with BAT response, including lower circulating PSA, decreased tumor volume, and higher overall survival (OS). IHC data showed that c-MYC was decreased in patients who responded to BAT. Experiments using PC cell lines showed that SPA, acting through the AR, decreased c-MYC and reduced cell growth, although other AR-regulated factors in addition to c-MYC may also be involved.

Using PC patient databases (13–15), the authors identified ten canonical AR target genes and applied Mann-Whitney ranking to generate a signature score, which the authors termed ARA_MW_. When applied to the COMBAT-CRPC pretreatment sample RNA-Seq data, a high pretreatment ARA_MW_ score predicted clinical responses to BAT in the cohort of 15 patients, suggesting that ARA_MW_ can serve as a biomarker for BAT response (12) (Figure 1). No collinearity was found among the genes, and ARA_MW_ did not correlate with AR levels across patients.

Sena et al. also provide valuable patient data for other investigators to interrogate with respect to BAT response (12). For example, Qiu et al. (11) defined a pretreatment AR cistrome that predicts response to SPA in PC patient-derived xenografts (PDXs), but, interestingly, this signature does not contain canonical AR target genes nor does it overlap with ARA_MW_.

In addition to the paired pre- and on-BAT biopsies of metastases from patients with CRPC, the authors used PC cell lines and a CRPC PDX to explore possible mechanisms underlying BAT responsiveness. While PC cell line-based experiments showed that high levels of AR and AR activity were required for SPA response, as previously observed (3, 16), patient data demonstrated that AR activity, and not AR abundance, predicted BAT response (12). This finding may be explained by the fact that AR protein levels do not necessarily correlate with AR transcriptional activity due to different CRPC adaptation mechanisms, such as AR activating mutations, changes in AR coregulators, and expression of constitutively active AR variants.

### Molecular mechanisms of response and resistance to SPA and BAT.

Sena et al. demonstrated that SPA response in sensitive PC cell lines was at least partially dependent on an AR-mediated decrease in c-MYC (also referred to as MYC), which is highly expressed in PC. Further, a PDX that responded to SPA, as indicated by decreased tumor volume, showed decreased MYC. Similarly, patients who responded to BAT exhibited a larger decrease in MYC compared with nonresponders. Additionally, patients with decreased MYC also correlated with those that had higher AR activity before treatment (12). Although the mechanism through which SPA decreases MYC in patients is unknown, a recent report using PC cell lines demonstrated that AR decreases *MYC* transcription independently of AR chromatin binding. This reduction in the transcription of *MYC* involves coactivator redistribution and perturbation of the interaction between the *MYC* super enhancer at the *PCAT1* gene and the *MYC* promotor (17).

While SPA-mediated suppression of MYC is a leading proposed mechanism underlying BAT response, studies in preclinical models have implicated alternative mechanisms, including impaired DNA licensing, which is a process where high levels of ligand-stabilized AR bind to the origins of DNA replication and promote early arrest in S-phase (18). Additional processes include induction of apoptosis and promotion of senescence (3). Since SPA produces AR-mediated DNA double-strand breaks leading to halted cell growth in several PC models, a potential vulnerability to PARP1 inhibitors may exist (10, 19). While the authors did not identify BAT-mediated alterations in the expression of a panel of homologous recombination repair–related (HRR-related) genes in the patient samples (12), an ongoing clinical trial combining BAT with the PARP1 inhibitor olaparib (NCT03516812) will address this important issue.

Alterations in androgen metabolism can accompany PC progression, including tumor switching from testosterone to androstenedione as the preferred precursor for DHT production (20). Additionally, the common germline variant 1245A>C in the enzyme encoded by *HSD3B1* leads to increased intratumoral DHT levels and promotes resistance to ADT and abiraterone treatment (21). This resistance mechanism is independent of changes in AR, and patients with this variant may not benefit from BAT treatment. Although it remains unknown whether alterations in androgen metabolism affect BAT response, a recent clinical trial (RESTORE-NCT02090114), showed that patients whose cancer progressed on enzalutamide had better response to BAT (PSA_50_ response and longer progression free survival 2 (PFS-2)) than men whose cancer had progressed on abiraterone (8).

## Clinical implications

While initial results from BAT clinical trials have been promising, most metastatic CRPC patients will develop secondary resistance after approximately 6–12 months of therapy (6). This publication and others suggest that resistance to SPA treatment is mediated by changes in levels and activity of AR (11). It is well known that AR is autoregulated—ligand-activated AR typically downregulates *AR* gene transcription, and, conversely, androgen stabilizes AR protein levels (22–26). SPA may perturb normal AR autoregulatory processes. Sena et al. reported that SPA eventually resulted in decreased levels and activity of AR as well as loss of MYC suppression, leading to treatment resistance in a PC cell line (12). Interestingly, treatment of the derived isogenic SPA-resistant PC cell line with the potent AR antagonist enzalutamide upregulated AR, which resensitized cells to SPA. This finding led the investigators to test alternating SPA with enzalutamide to produce extreme oscillations in AR activity in the CRPC PDX model. Castrated mice treated with this SPA/enzalutamide paradigm did not exhibit resistance for the duration of the experiment (160 days) (12). Additionally, tumors from this SPA/enzalutamide arm had lower levels of MYC than those from mice that only received SPA (12). The cycling of BAT with ARSIs may provide additional benefits to patients and is one of the hypotheses of the upcoming STEP-UP clinical trial (NCT04363164).

To date, approximately 350 patients with metastatic CRPC were treated with BAT in three phase II trials (TRANSFORMER, RESTORE, and COMBAT) (9). Those trials demonstrated that BAT (a) can be safely given to asymptomatic patients, (b) is moderately effective, and (c) has the potential to resensitize CRPC to subsequent therapy with ARSIs. As discussed above, the vast majority of metastatic CRPC patients will eventually develop secondary resistance to BAT (6). To improve the efficacy and duration of response, ongoing studies are evaluating treatment combinations of BAT with the immune checkpoint inhibitor Nivolumab (NCT03554317), the DNA-repair inhibitor Olaparib (NCT03516812), the bone-targeted radiation therapy Radium 223 (NCT04704505), and carboplatin (NCT03522064).

## Conclusion

The Sena et al. study identified high AR activity as a requirement for clinical response to BAT, which is associated with downregulation of MYC. ARA_MW_ is a potential biomarker that may stratify responders from nonresponders; however, findings should be validated in an independent patient cohort. Although BAT therapy has the potential to improve the outcome of patients with metastatic CRPC while preserving good quality of life, treatment is not risk free and does not work for all patients. Therefore, BAT should not be used outside of a clinical trial, especially in combination with other therapies. Larger and randomized clinical studies are needed in the future to validate and determine the best way to integrate BAT into metastatic CRPC treatment algorithms.

## Version 1 12/01/2022

Electronic publication
